# Role of Cardiovascular Magnetic Resonance to Assess Cardiovascular Inflammation

**DOI:** 10.3389/fcvm.2022.877364

**Published:** 2022-07-06

**Authors:** Domenico Filomena, Tom Dresselaers, Jan Bogaert

**Affiliations:** ^1^Department of Imaging and Pathology, KU Leuven, Leuven, Belgium; ^2^Department of Radiology, University Hospitals Leuven, Leuven, Belgium

**Keywords:** cardiovascular magnetic resonanace, inflammation, imaging, myocarditis, pericarditis, vasculitis

## Abstract

Cardiovascular inflammatory diseases still represent a challenge for physicians. Inflammatory cardiomyopathy, pericarditis, and large vessels vasculitis can clinically mimic a wide spectrum of diseases. While the underlying etiologies are varied, the common physio-pathological process is characterized by vasodilation, exudation, leukocytes infiltration, cell damage, and fibrosis. Cardiovascular magnetic resonance (CMR) allows the visualization of some of these diagnostic targets. CMR provides not only morphological and functional assessment but also tissue catheterization revealing edema, hyperemia, tissue injury, and reparative fibrosis through T2 weighted images, early and late gadolinium enhancement, and parametric mapping techniques. Recent developments showed the role of CMR in the identification of ongoing inflammation also in other CV diseases like myocardial infarction, atherosclerosis, arrhythmogenic and hypertrophic cardiomyopathy. Future developments of CMR, aiming at the specific assessment of immune cell infiltration, will give deeper insight into cardiovascular inflammatory diseases.

## Introduction

Inflammation plays a key role in most cardiovascular diseases. However, the term inflammatory cardiovascular (CV) diseases refers to an abnormal inflammation of the myocardium, pericardium, or large vessels which is the primary driver of the disease (1). Cardiovascular magnetic resonance (CMR) plays a key role in the assessment of cardiovascular inflammation allowing not only functional assessment but also tissue characterization (2).

### Inflammatory Cardiomyopathy

Myocarditis is pathologically defined by the presence of inflammatory infiltration of the myocardium associated with degenerative and/or necrotic changes of cardiomyocytes not due to ischemia/infarction (3, 4). The most common etiology is a viral infection, while other pathogens (bacteria, protozoa, and fungi) are less frequently involved (5). Drugs/toxic exposures and systemic or organ-specific autoimmune disorders can also cause myocarditis (6). The etiological cause determines the exposure of specific molecular patterns [either pathogen-associated molecular patterns (PAMPs) or damage-associated molecular patterns (DAMPs)] that activate the innate immune system. Subsequently, the latter triggers the acquired immune response with T- and B-cell proliferation (7). The inflammatory cell infiltration can significantly vary, encompassing lymphocytes, phagocytes, multinucleated giant cells, and/or eosinophil cells. Complex non-caseating granulomas may be a signature of a specific inflammatory pathway (7). The persistence of the immune activation leads to a different grade of cell degeneration and/necrosis. In a wide sense under the category of inflammatory cardiomyopathy several diseases are reunited: lymphocytic viral myocarditis, HIV cardiomyopathy, connective tissue disease, giant cell myocarditis, cardiac sarcoidosis, Chagas cardiomyopathy, hypersensitivity myocarditis, Löffler endocarditis, and endomyocardial fibrosis (8). A detailed description of the aforementioned diseases goes beyond the purpose of this review. A specific form of inflammatory myocardial disorder is allograft rejection after heart transplant driven by allo- and auto-immunity activation (9).

### Pericarditis

Acute pericarditis is an inflammatory pericardial syndrome with or without pericardial effusion. Several etiologies are shared with the myocardial inflammatory diseases (infectious, toxic, and auto-immune), while *Mycobacterium tuberculosis*, metabolic diseases, and neoplasm are additional and not-infrequent causes of pericardial inflammation (10). In a non-negligible number of cases, a definite etiology cannot be found. While they are frequently defined as idiopathic, an underlying viral infection or an autoimmune cause can be generally suspected. Post-cardiac injury syndromes are another under-diagnosticated form of pericarditis caused by an immune response to different types of myocardial, epicardial, and pericardial injury (11).

### Vasculitides

The term “large vessel vasculitides” encompasses a wide spectrum of inflammation of the great arteries. The most common causes are giant cell arteritis and Takayasu arteritis characterized by granulomatous inflammations. Overlap with other forms of systemic vasculitides is possible and inflammation of great arteries can be found in Kawasaki disease, Behçet syndrome, rheumatoid arthritis, and IgG-4 related disease. Infectious vasculitides are less frequent but syphilis and tuberculosis are possible etiology which diagnosis should prompt specific treatments.

### Endocarditis

While inflammation is involved in the pathogenesis of infectious endocarditis, this nosological entity is not frequently classified among cardiovascular inflammatory diseases. However, infectious endocarditis should be considered in the differential diagnosis especially when interpreting nuclear imaging. On the other hand, endocardial involvement can be found in different inflammatory diseases: eosinophilic cardiomyopathy (Löffler endocarditis and endomyocardial fibrosis), Libman–Sacks endocarditis, marantic endocarditis, and rheumatic endocarditis (12).

## Physiopathology in CV Inflammation

Inflammation is a stereotyped response of body tissue to harmful stimuli (pathogens and/or damaged cells) whose objective is to eliminate the insult and repair the tissue. It involves both innate and cell-mediated immune responses (13). Both tissue injury and pathogen molecular patterns are recognized by resident immune cells which release or activate chemical mediators (histamine, kinins, prostaglandins, and complements). They are responsible for vasodilatation, increase capillary permeability, exudate formation, chemotaxis, and leukocyte transmigration. The ultimate effects are hyperemia, edema, leukocyte infiltration. The activation of specific pathways of cellular immunity and cell-derived mediators determine different subtypes of inflammation (e.g., lymphocytic, eosinophilic, granulomatous) (13). Pathogens and the phlogistic process itself concur to determine the various grade of tissue injury and cell necrosis. Possible outcomes of the inflammatory process are complete resolution, fibrosis and scarring, and chronic inflammation (14). Because of acute inflammation or the reparative processes, different grades of tissue/organ dysfunction (functio lesa) can be observed. Both exudate formation, necrosis, and fibrosis concur in expanding extracellular volume (ECV).

Some of the aforementioned pathophysiological characteristics of phlogosis are used by CMR to diagnose inflammatory cardiovascular diseases.

## Cardiovascular Magnetic Resonance Biomarkers in CV Inflammation

**FIGURE 1:**
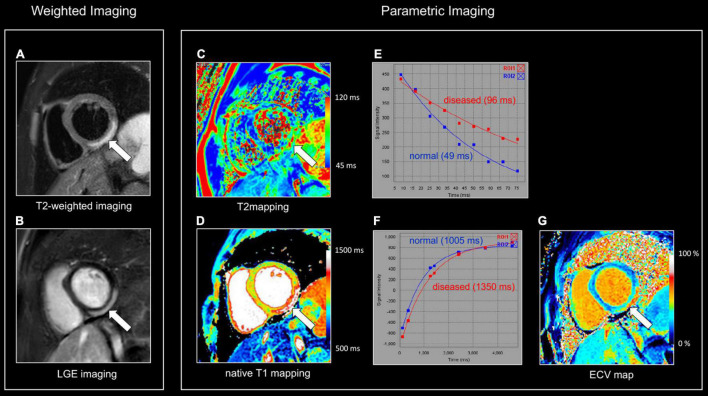
Myocardial tissue characterization using comprehensive CMR in myocardial inflammation. Sixteen-year-old boy admitted with fever and acute chest pain, presenting negative Q-waves and ST elevation in the inferior leads at ECG. Elevated high-sensitivity cardiac troponin T (hs cTnT). Transthoracic echocardiography shows area of akinesia in mid inferior left ventricular (LV) wall and small pericardial effusion. Normal coronaries at cardiac computed tomography. A combination of “weighted” **(A,B)** and parametric (T1/T2 mapping) CMR sequences was performed **(C,D)**. All images are obtained in cardiac short-axis. As T1-weighted imaging provides limited information in myocardial tissue characterization it is not routinely performed. In contrast, T2-weighted imaging **(A)** shows subepicardial rim of hyperintense (“bright”) signal in the inferolateral LV wall [arrow, **(A)**], reflecting myocardial edema. Post-contrast, i.e., late gadolinium enhancement (LGE), imaging **(B)** shows strong uptake of contrast [arrow, **(B)**], closely corresponding to the region of myocardial edema at T2-weighted imaging. Parametric T2 map (Royal color scheme, range 45–120 ms) **(C)** and T2 relaxation curve **(E)** in the normal and diseased myocardium shows prolonged T2 relaxation times in the edematous myocardium (i.e., 96 vs. 49 ms). Parametric native T1 map (Royal color scheme, range 500–1,500 ms) **(D)** and T1 relaxation curve **(F)** show prolonged T1 relaxation times in the pathologic myocardium (1,005 vs. 1,350 ms). Finally, the extracellular volume (ECV) map **(G)** shows increased values [arrow, **(G)**] in the diseased myocardium (i.e., 45%) versus 24% in the normal myocardium.

**FIGURE 2:**
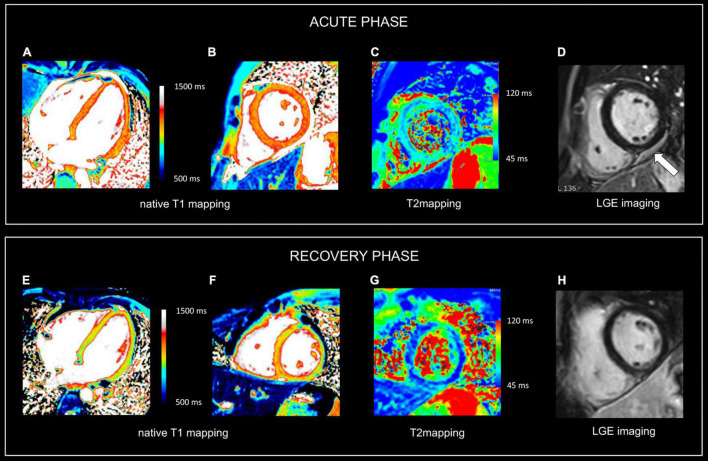
Use of CMR in acute and follow-up phase of diffuse myocarditis. Sixteen-year-old boy admitted with viral myocarditis and heart failure. Increased troponin I (12,239 ng/L), NTproBNP (23,731 ng/L) and c-reactive protein (183 mg/L). At admission, transthoracic echocardiography shows non-dilated but severely dysfunctional LV (EF 20%) and mild pericardial effusion. CMR, performed five days later, shows almost complete functional recovery (i.e., LV ejection fraction 57%). Although LGE imaging **(D)** shows no focal myocardial abnormalities, native T1, T2 and ECV values are diffusely increased, i.e., 1125 ms, 69 ms and 34% [panels **(A–C)**, respectively]. Presence of limited pericardial effusion [arrow, **(D)**]. Repeat CMR 4 months later (recovery phase) shows normalization of T1, T2 and ECV values, i.e., 975 ms, 51 ms, and 24% [panels **(E–G)**, respectively], while LGE imaging **(H)** yields no evidence of permanent irreversible damage (i.e., focal replacement fibrosis). Moreover, a further improvement in LVEF was noted, i.e., 64% with decrease in LV mass (i.e., 83 vs. 102 g at baseline), and disappearance of the pericardial effusion. In the clinical setting, the baseline CMR findings most likely represent diffuse myocardial edema/inflammation caused by viral myocarditis. As the myocardium is diffusely affected, weighted sequences, i.e., T2 and LGE imaging are “normal” emphasizing the added value of parametric mapping in these patients.

**FIGURE 3:**
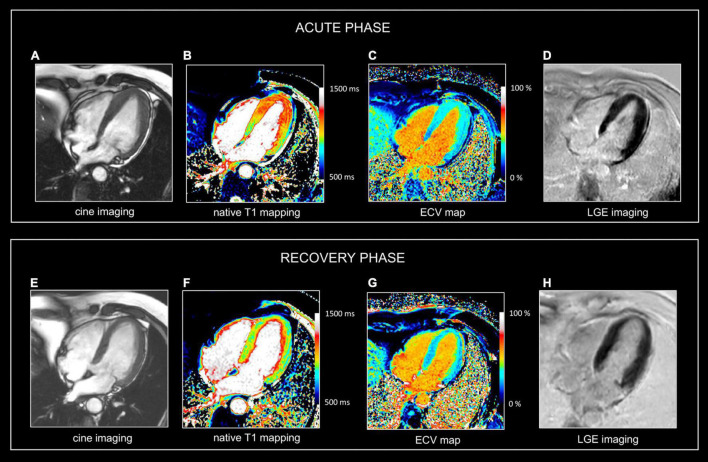
Use of CMR in acute and follow-up phase of focal myocarditis. Sixty-nine-year-old man presenting with fatigue, dyspnea (NYHA II), presyncope. ECG showed anterior repolarization disturbances. Increased release of hs cTnT (0.033 μg/L). No significant coronary artery disease at cardiac catheterization. CMR at admission **(A–D)** and at 1-year follow up **(E–G)**. At baseline, there is a marked myocardial swelling (14 mm) of the apical half of the left ventricle **(A)**, showing increased native T1 values **(B)**, T2 (i.e., 63 ms (not shown) and increased ECV values **(C)** but no LGE **(D)**. Preserved LV ejection fraction (i.e., 71%) but moderately decreased global longitudinal strain (i.e., -13.2%). Presence of a mild pericardial effusion. At one-year follow-up, the LV wall thickness as well as the T1, T2, and ECV values normalized in LV apex. Global longitudinal strain improved (i.e., -16.3%), and the mild pericardial effusion disappeared. No abnormal LGE **(H)**. In summary, CMR shows transient myocardial edema/inflammation in LV apex, however, of unknown etiology. Overall, findings are most suggestive of focal myocarditis completely recovery at follow-up. Although similar abnormalities can be found in patients with takotsubo cardiomyopathy, the lack of functional deficit in the LV apex is not in favor of this diagnosis. Also, an aborted myocardial infarction with extensive myocardial edema is not very likely because of the lack of coronary artery disease at cardiac catheterization and the absence of an ischemic pattern of involvement (i.e., abnormalities not spread along a coronary artery perfusion territory).

Cardiovascular magnetic resonance is typically comprehensive or multi-parametric, which means that a set of CMR sequences is selected to answer as appropriately as possible on the clinical question (Figure 1). Each of these sequences has been optimized to study or to visualize one specific feature of the cardiovascular system (Table 1). Combining this sequence-specific information usually enables to achieve an accurate view on the presence, severity, location and extent of a CV inflammatory process, to assess the functional consequences, and to look for complications (e.g., aneurysm formation in endocarditis patients). This approach makes CMR, compared to other (imaging) methodologies, quite unique. Moreover, thanks to its non-invasive nature, CMR is ideal to follow-up patients with known, or that are at risk of developing, CV inflammation (Figures 2, 3).

**TABLE 1:** Strengths, weaknesses, and applications of current CMR sequences to study cardiovascular inflammation.

CMR sequences	Strengths	Weakness	Applications
**B-SSFP cine images**	High signal-to-noise ratio High spatial and temporal resolution	Artifact in case of ECG-mistriggering and poor breath-hold	Morphological assessment of heart and great vessels Ventricular volumes and systolic fuction assessment (including strain calculation)
**Real time cine images**	No need for ECG-trigger and breath-hold	Lower signal-to-noise ratio Lower spatial and temporal resolution	Ventricular interdependence evaluation
**Black-blood T1-weighted images**	High spatial resolution	Static images: no dynamic information	Morphological assessment of heart and great vessels: particularly, pericardial and vascular wall thickness evaluation
**T2-weighted images**	Widely available Detection of focal increase in tissue water content	Low signal-to-noise ratio Slow flow artifacts. Possible insufficient fat suppression and field inhomogeneity artifacts. Unable to image diffuse edema	Detection of myocardial, pericardial and vascular wall edema (hyperintensity)
**Early gadolinium enhancement**	Widely available Helpful when integrating LGE and T2W images for focal disease	Affected by image timing after contrast administration. Unable to detect diffuse disease	Detection of myocardial hyperaemia and increased vascular permeability (hyperintensity)
**Late gadolinium enhancement**	Widely available Easy differentiation between ischemic and non-ischemic pathology	Unable to detect diffuse disease Artifact in case of ECG-mistriggering, poor breath-hold and poor myocardial nulling (overcome using several available LGE sequences)	Detection of focal tissue injury, necrosis and replacement fibrosis (hyperintensity)
**Parametric imaging**	Quantification of T1 and T2 relaxation times Extracellular volume quantification Able to detect diffuse disease	Less widely available T1 and T2 relaxation times affected by scanning conditions (need for center-specific reference values) Utility limited to the myocardium	Detection of myocardial edema: increased T1, T2 and ECV Detection of myocardial hyperemia: increased T1 and/or ECV Detection of myocardial fibrosis: increased T1 and ECV
**MR angiography**	Three-dimensional angiographic reconstruction of great and medium size vessels	No information regarding to the vessel wall	Detection of vessel stenosis and/or aneurysm

*b-SSFP, balanced steady-state free precession; CCT, cardiac computed tomography; ECV, extracellular volume; GBCA, gadolinium-bound contras agent; ICD, implantable cardioverter defibrillator; MIP, maximum intensity projection; MPI, myocardial perfusion imaging; MPR, multiplanar reconstruction; MRA, magnetic resonance angiography; PC, phase-contrast; SE, spin-echo; SPECT, single photon emission computed tomography; VR, volume-rendered.*

### Morphological and Functional Imaging

Cardiovascular magnetic resonance has become the “gold standard” for morphological and functional assessment of the cardiovascular system. Balanced steady-state free precession (b-SSFP) sequences, combining high spatial and temporal resolution with high blood/tissue contrast, allow kinetic visualization of the myocardium and great vessels (2, 15). Assessment of cardiac volumes and systolic function alongside morphological evaluation of the pericardium and great arteries are the main uses of this technique (16). Thanks to recent progress in machine learning algorithms, quantification of cardiac volumes and function has been (semi)-automated (17). More sensitive parameters of (early) cardiac dysfunction, such as impaired myocardial strain, can be derived using feature tracking techniques and CMR tagging sequences (18). The latter can be applied as well to visualize the shear motion between pericardial layers in patients with inflammatory or constrictive pericarditis. In patients with metallic implants a spoiled gradient echo sequence can be beneficial to reduce magnetic susceptibility artifacts as may occur with b-SSFP. In case of poor breath holding free breathing real-time (RT) techniques can be applied (15). RT sequences also enable to study dynamic, respiratory-determined physiologic events, as ventricular coupling (19). Finally, black-blood T1-weighted (T1w) spin-echo CMR is the preferred approach to “statically” visualize the heart, pericardium, great vessel, and mediastinum (20). In particular, it is an excellent sequence to visualize the pericardial thickness and to image the vessel wall, which is for example highly useful in patients with pericarditis or aortitis.

### T2-Weighted Images

T2-weighted (T2w) spin-echo images are extremely useful to evaluate tissue edema which is visualized as a hyperintense (“bright”) signal (21). Several T2w approaches are currently available. Historically, a short tau inversion recovery (STIR) technique was used to suppress signal of blood and surrounding epicardial and mediastinal fat. Possible pitfalls of STIR T2-weighted imaging include surface coil reception field inhomogeneity, high SI artifacts due to slow-flowing blood with consequent insufficient flow suppression and signal loss due to through-plane cardiac motion during the black blood preparation, often most noticeable in the posterior wall. As an alternative, a frequency selective fat suppression can be used to overcome some of the above-mentioned limitations (21). Moreover, as visualization of tissue edema by T2w imaging relies on the differences in signal intensity (SI) between normal and edematous tissue, focal (myocardial) edema is well depicted, while diffuse myocardial inflammation may be missed (Figure 1A). Quantitative parametric T2 mapping, in contrast, allows to measure T2 relaxation times irrespective of the extent of tissue edema (22).

### Early Gadolinium Enhancement (EGE)

Gadolinium-based contrast agents (GBCA) are paramagnetic extravascular and extracellular contrast agents whose use is a cornerstone of cardiovascular magnetic resonance (15). Its accumulation in tissues shortens both T1 and T2 relaxation times. Hyperemia, increased vascular permeability and increased extracellular volume in the acute inflammatory process lead to an enhanced early accumulation of GBCA in inflamed tissue compared to the normal one. Depiction of hyperemia, however, is still challenging. Older techniques, such as conventional T1 weighted spin-echo imaging have been abandoned (23), while novel approaches such as early T1 mapping need further investigation (24).

### Late Gadolinium Enhancement

Beyond edema, a further increase in extracellular space is due to the various grades of tissue injury with necrosis and fibrosis occurring during, or as a consequence, of inflammatory processes. In case of necrosis, GBCA can enter in injured cells, significantly increasing its distribution territory. T1w images late after contrast administration are used to image tissue enhancement, i.e., late (or delayed) gadolinium enhancement (LGE). Rather than using fast T1w spin-echo images, a balanced SSFP or GRE sequence is used in combination with an inversion-recovery prepulse. The latter is aimed to suppress the signal of normal tissue (e.g., myocardium) by selecting a correct inversion time length, and thus to increase the difference in contrast between normal and pathologic myocardium (Figure 1B) (25). Several sequence variants are available with different strengths and limits: standard or phase sensitive inversion recovery (PSIR) with myocardial or blood-nulling, breath-hold or respiratory-gated, 2D and 3D acquisition (26). LGE should be confirmed in two different orthogonal planes and in case of uncertainty, it should persist after changing the phase encoding direction (16). Some challenging situations are still possible such as the differentiation with septal perforator coronary artery, adjacent tissue with high T1 signal in non-PSIR sequences or in case of a very thin myocardium. Patterns of LGE distribution differentiate ischemic- from non-ischemic myocardial disease (subendocardial vs non-subendocardial LGE distribution, respectively) (27).

### Parametric Imaging

While depiction of diseased tissue using ‘weighted’ MRI sequences [i.e., T1w, T2w, early gadolinium enhancement (EGE) and LGE] relies on differences in SI with the surrounding, deemed normal, tissues, mapping techniques allow for the reconstruction of parametric maps of both T1 and T2 relaxation times on a pixel-by-pixel basis (Figures 1C–F, 2) (28). Consequently, drawing a region of interest (ROI), calculation of the local T1 and T2 relaxation times is possible. An in depth, discussion on the different T1 and T2 mapping sequences is beyond the scope of this review paper. However, it is important to understand that T1 and T2 relaxation times are also influenced by the magnetic field strength (both increase with higher field strength) and by the type of sequence used (29). These are important issues when interpreting T1 and T2 values, as they are not trivially interchangeable between machines/vendors/sequences. As most of the influencing factors can be standardized, age- and gender-corrected normal values (and cut-off values) for T1 and T2 can be determined, offering a truly quantitative approach on tissue characterization (30, 31). Thus, using similar scanning conditions, T1 and T2 relaxation times can be used to monitor disease progression (Figures 2, 3).

As mentioned above, the pixel-by-pixel relaxometry overcomes some limitations of SI-based techniques allowing the evaluation of both focal and diffuse tissue pathology. T2 Mapping—which is always acquired before contrast administration – is highly sensitive for tissue water (32). Abnormally increased T2 values reflect focal or diffuse tissue edema. Parametric T1 mapping is acquired before (native T1) and often after contrast administration. Native T1 values can be increased or decreased. The latter is seen in conditions with increased lipid or iron content such as Fabry’s disease or hemochromatosis. Also, in focal diseases such lipomatous metaplasia post myocarditis, and in hemorrhagic myocardial infarctions, native T1 values are lower in the affected tissue (33). Native T1 values are increased in several conditions, and therefore should be considered a non-specific biomarker as it may reflect edema, necrosis, fibrosis, and interstitial deposition of abnormal materials (e.g., amyloid) (34, 35). Ideally, native T1 values should be interpreted together with T2 values, as increased T2 values normally are related to increased native T1 values (Figures 2, 3). In contrast, increased T1 values with normal T2 values of the myocardium make myocardial edema/inflammation not very likely but instead may be encountered for example in patients with cardiac amyloidosis. Moreover, information regarding the extracellular volume (ECV) can be obtained by repeating T1 mapping 10–20 min after GBCA administration. Abnormal, i.e., increased, accumulation of GBCA results in a higher T1 shortening. By measuring both native and post-contrast T1 mapping values of the myocardium and blood compartment as well as the hematocrit, the ECV can be calculated (30). Like native T1 and T2 values, normal ECV values can be calculated. In summary, parametric mapping has revolutionized our look at myocardial disease, and rapidly shown indispensable in addition to LGE imaging. However, it should be noted parametric mapping is currently of limited value to assess thin structures such as the right ventricular wall, the atria, the pericardium or vessel wall.

### Magnetic Resonance Angiography

**FIGURE 4:**
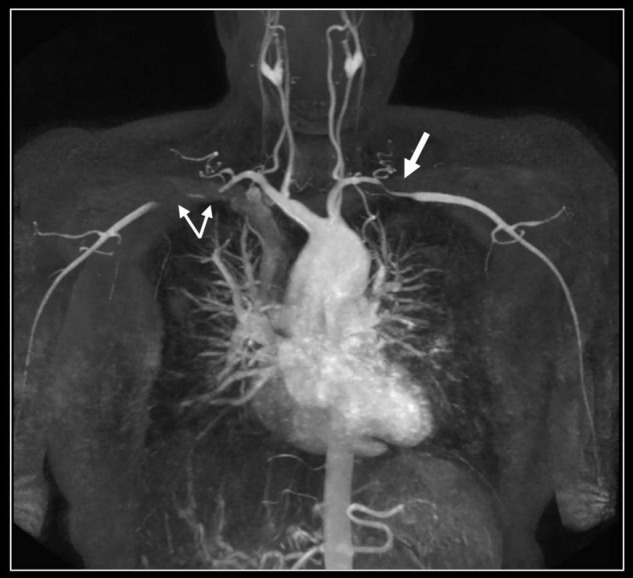
Contrast-enhanced 3D MR angiography. Forty-year-old woman known with Takayasu disease treated with steroids and immunosuppressive therapy (Imuran). At coronal MIP a focal high-grade narrowing of the right subclavian (thin arrows) and less severe stenosis of the left subclavian coronary artery (thick arrow) can be well appreciated. No evidence of aneurysm formation of the thoracic aorta (ascending aorta 32 mm) nor of the side branches.

**FIGURE 5:**
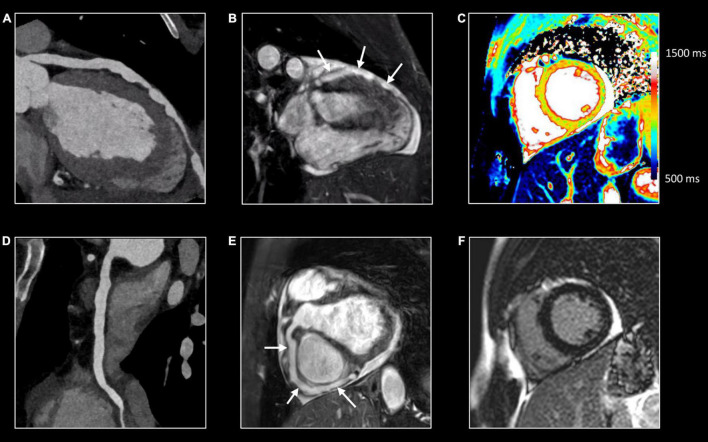
Comprehensive imaging in coronaritis. Twenty-seven-year-old man with systemic vasculitis, likely adult-onset Kawasaki disease), admitted with non-STEMI. Computed tomography (CT) **(A,D)** and CMR **(B,E)** of the coronary arteries show irregular aneurysmatic dilation (up to 8 mm) of the coronary arteries with on the left anterior descending (LAD) coronary artery several skip areas (focal pseudostenoses). Although the CMR was requested because of clinical suspicion of non-STEMI, CMR revealed no myocardial edema (normal T1/T2 mapping) **(C)** nor abnormalities at LGE imaging **(F)** suggestive of myocardial necrosis and/or fibrosis. Further investigation showed a normal caliber of the thoraco-abdominal aorta. However, aneurysms were found on several middle-sized abdominal arteries, including the celiac trunk, common hepatic artery, superior and inferior mesenteric artery and both renal arteries.

Both non-contrast and contrast-enhanced 2D/3D sequences are available for magnetic resonance (MR) angiography (36). Balanced SSFP images, with a high spatial and temporal resolution, allow great vessel assessment in different planes. Trans-axial and parasagittal acquisitions provide the required information in most cases. A 3D angiographic reconstruction is possible after contrast administration imaging during the first pass of the contrast in the vessel of interest. Alternatively, time-resolved techniques are available if different vascular structures need to be visualized. 3D rendering, maximum intensity projection (MIP) and multiplane reconstruction (MPR) are used for post-processing and vascular assessment (Figure 4) (37). Also, for coronary artery imaging, several CMR strategies dealing—with the numerous imaging challenges intrinsically related to the visualization of these small vessels along the curved cardiac surface—are currently available. With the advent of advanced high-quality, coronary computed tomography, however, coronary artery imaging is not part of a routine CMR exam, but in selected cases may provide valuable information (Figure 5). Moreover, it is important to emphasize MR angiography sequences provide only a luminographic view allowing to depict stenosis and/or aneurysm formation but no information regarding the vessel wall (Figures 4, 5). Thus, in patients with vasculitis, T1 and T2-weighted spin-echo images are obligatory.

### Other CMR Sequences

Although the above sequences cover the entire spectrum of sequences to assess cardiovascular inflammation, in routine practice, flow imaging (2D/4D) using phase-contrast CMR is often used to quantify the severity of concomitant valvular regurgitation/stenosis, or to assess intravascular flow patterns in diseased vessels. Moreover, stress perfusion imaging may be indicated if myocardial ischemia is suspected.

## CMR in Inflammatory Cardiomyopathy

CMR is the method of choice for the evaluation of patients with clinically suspected myocarditis without hemodynamic instability (2, 38). The updated Lake Louise Criteria are the reference for myocardial inflammation diagnosis (39). Differently from the first version of the consensus, in the revised criteria a “2 out of 2” approach is used, with one positive T2-based criterion and one T1-based criterion considered necessary to increase the specificity of detecting acute myocardial inflammation. T2-based criteria use myocardial edema as a diagnostic target. Positive T2-based criterion is defined as the presence of regional or global increased SI at T2w imaging and/or regional or global increase of the myocardial T2 relaxation time using T2 mapping (Figures 1–3) (39–41). Conversely, positive T1-criterion is defined as a regional or global increase of the native myocardial T1 relaxation time or ECV and/or the presence of LGE with a non-ischemic distribution pattern (Figures 1–3) (42, 43). While an increased native T1 and ECV may reflect edema, hyperemia, necrosis, or fibrosis, the presence of LGE is more specifically related to myocardial injury with necrosis and reparative fibrosis (34, 42, 43). While not included in the revised criteria, the presence of EGE can be used in experienced centers especially in cases of non-diagnostic T2W or LGE images (24). Global or regional systolic dysfunction can be detected but frequently, the limited extension of the inflammation and the counteracting hypercontractility of adjacent segments can result in normal systolic function (44). The presence of pericardial effusion or edema is considered a supportive criterion (Figure 2). Myocarditis involves most frequently the inferolateral LV wall, although any segment of the heart can be affected (Figure 1) (43). The diagnostic performance of these criteria has been demonstrated to be high, with better results in the infarct-like presentation of acute myocarditis (45, 46). Diagnostic performance of Lake-Louise criteria is lower when assessing patients with chronic, heart failure-like symptoms, and in differentiating inflammatory vs other forms of non-ischemic cardiomyopathy. In these cases, only the T2 criterium showed acceptable diagnostic performance (22). Moreover, in the case of diffuse or chronic ongoing myocarditis, as in the certain form of allotransplant rejection, SI-based techniques may fail to determine the presence of inflammation while parametric techniques may unmask diffuse edema and/or interstitial fibrosis (Figure 2) (42).

**FIGURE 6:**
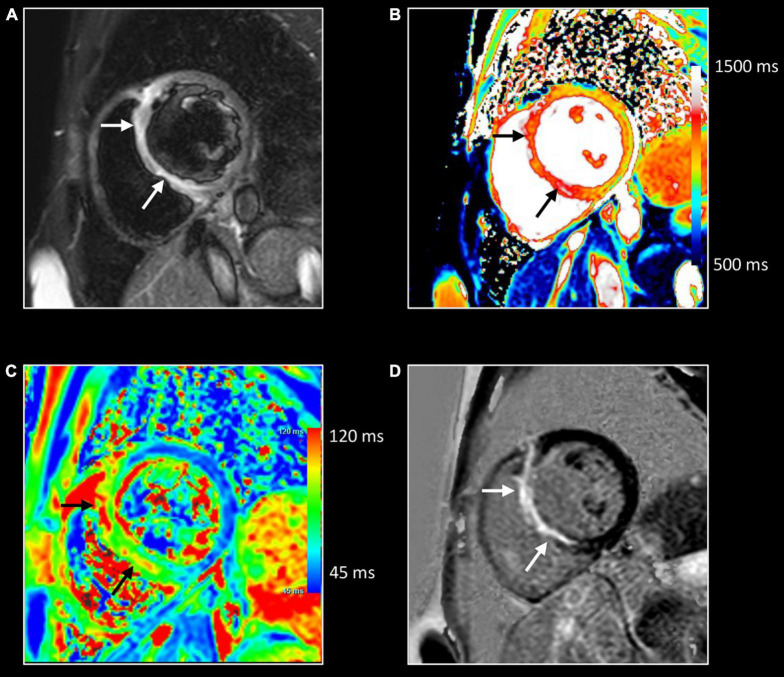
Giant cell myocarditis rapidly evolving to heart failure. Forty-six-year-old woman presenting with monomorphic ventricular tachycardia arising from the basal septum. Transthoracic echocardiography shows mildly decreased LV function (EF 49%) with dyskinetic basal septum. CMR shows mildly dilated and dysfunctional left ventricle (LVEDVi 107 ml/m2 – LVEF 45%), and thinned, dyskinetic basal septum. T2w-imaging **(A)** shows myocardial edema in the basal part with strong increase of native T1 (i.e., 1,230 ms) and T2 (i.e., 99 ms) relaxation times in this area [arrows **(B,C)**, respectively]. Strong uptake of contrast showing a non-ischemic enhancement pattern but in a large extent completely transmural [arrows, **(D)**] and increased ECV (i.e 65%) (not shown). Findings suspected of active inflammation in the basal septum with local adverse remodeling. A left-sided myocardial biopsy showed giant cell myocarditis. Follow-up CMR, one month later, showed important deterioration of LV function (LV EF 26%). Because of the unfavorable outcome, the patient received a cardiac transplant. Two years later, she is doing well.

Acute myocarditis can heal with complete “restitutio ad integrum” but frequently focal or multifocal fibrosis can be detected at LGE, most commonly depicted subepicardially in the inferolateral LV wall (44). A possible outcome of myocardial inflammation is the development of myocardial systolic dysfunction with or without left ventricular dilation, frequently indistinguishable from dilated cardiomyopathy phenotype (5). The evaluation of the presence of myocardial scarring, even in a functionally normal heart, has prognostic information, especially related to arrhythmic consequences of myocarditis (44, 47). The extent and location of the LGE seem to affect prognosis in patients with myocarditis. More extensive LGE and anteroseptal locations were found associated with major adverse cardiac events (Figure 6) (48, 49).

**FIGURE 7:**
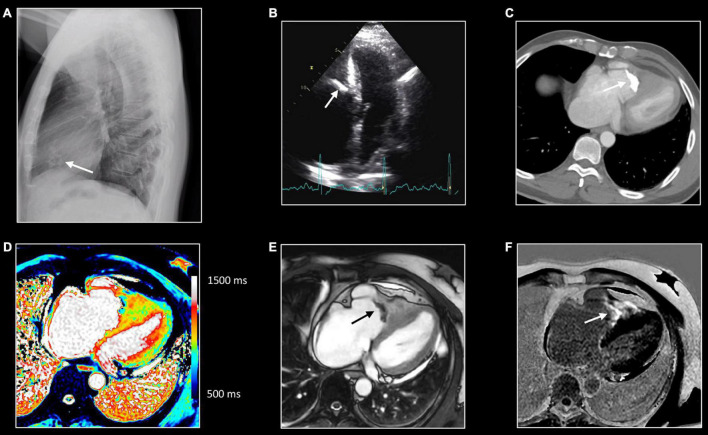
Right sided endomyocardial fibrosis. Sixty-seven-year-old man with extensive fibro-calcification of the right ventricular apex, severe tricuspid regurgitation, and dilatation of the right atrium, caval and hepatic veins. Lateral chest x-ray **(A)** shows amorphous calcifications (arrow) in the anterior half of the cardiac silhouette, which is confirmed at transthoracic echocardiography [arrow, **(B)**] as well on computed tomography (CT) [arrow, **(C)**]. Native T1 mapping **(D)**, post contrast bSSFP cine imaging **(E)** and LGE imaging **(F)**. All CMR images were acquired in horizontal long-axis. The obliteration of the right ventricular cavity by the fibrocalcific process can be well appreciated at CT and CMR. Native T1 mapping shows inhomogeneous appearance with strong enhancement at LGE imaging, reflecting extensive replacement fibrosis [arrow, **(F)**].

CMR usually fails to identify the etiology of the inflammatory process. However, certain subtypes of myocardial inflammatory disease have peculiar characteristics. The role of CMR in giant cell myocarditis is of limited importance due to the dramatically rapid evolution of this inflammatory disease (Figure 6). Diagnosis is more frequently made by endomyocardial biopsy. In limited experiences, CMR showed large areas of edema and necrosis, frequently with atypical sub- endocardial involvement (50). Diffuse subendocardial layer involvement has been shown also in eosinophilic cardiomyopathies including cardiac involvement of eosinophilic granulomatosis with polyangiitis (51). Thickening and fibrosis of the endocardium characterize Löffler endocarditis and endomyocardial fibrosis with endocardial and subendocardial fibrosis (Figure 7). Endocardial involvement is frequently accompanied by thrombus formation and apex obliteration of the ventricle. Right ventricle involvement is described in up to 50% of cases (52). Functionally, these entities are characterized by restrictive physiology with low left ventricular volumes, preserved systolic function, and signs of increased diastolic pressure with marked left and right atrial enlargement. Involvement of valvular endocardium is possible with thickening and thrombosis.

**FIGURE 8:**
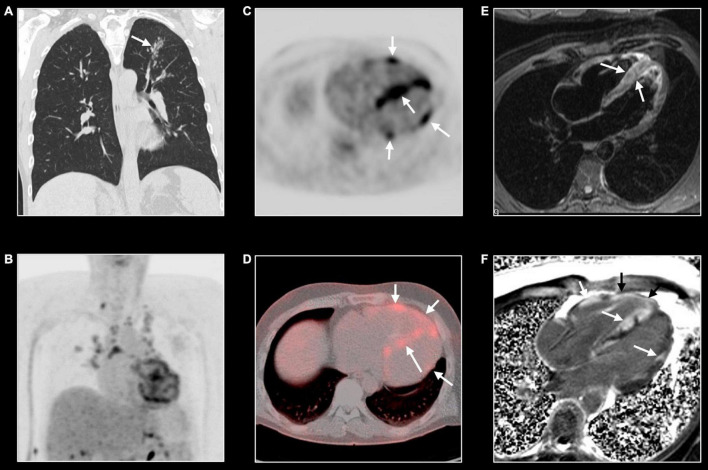
Cardiac sarcoidosis. Forty-three-year-old man known with systemic sarcoidosis admitted with recurrent ventricular tachycardia. Chest CT **(A)** shows enlarged mediastinal lymph nodes and perilymphatic micronodular pattern in left upper lobe (arrow), right middle and lower lobe. Fluoro-deoxy-gluclose (FDG) PET/CT shows multifocal FDG uptake in mediastinal and hilar lymph nodes **(B)**, and strong multifocal uptake in the myocardium of left and right ventricle [arrows, **(C,D)**]. T2w-imaging shows myocardial edema, most pronounced in the apical half of a thickened ventricular septum [arrows, **(E)**] while LGE imaging shows multifocal, pronounced myocardial enhancement in left and right ventricle [arrows, **(F)**]. CMR and PET/CT findings are strongly suggestive of cardiac sarcoidosis with severe biventricular involvement. Cardiac biopsy showed granulomatous myocarditis. Because of the cardiac arrhythmias, the patient received an ICD implantation with several appropriate shocks. Unfortunately, the patient evolved toward biventricular heart failure for which he underwent a heart transplantation. Eight years later, the clinical history is uneventful.

Cardiac involvement has been described in almost 25% of systemic sarcoidosis (53). CMR features are non-ischemic multifocal “patchy” myocardial LGE, reflecting diffuse infiltration by sarcoid granulomas (Figure 8). However, there is no specific LGE pattern considered pathognomonic for cardiac sarcoidosis (54, 55). In the early phase of the disease, only inflammation and mild myocardial thickening can be detected. In the late phases of the disease thinning of the myocardium, especially in the basal interventricular septum, and systolic dysfunction can be seen. The presence of LGE has been shown to have prognostic relevance related to the arrhythmic outcome of patients with cardiac sarcoidosis (56). Chagas cardiomyopathy, a specific subtype of infective myocarditis, is characterized by apical LV aneurysm with concomitant LGE, with or without thrombus formation, and various grades of systolic dysfunction (57). Myocardial involvement can be observed in patients with autoimmune rheumatic diseases, increasing both morbidity and mortality. Particularly, myocardial abnormalities are frequent in patients with systemic sclerosis. In those patients increased T1 and ECV values, reflecting diffuse interstitial fibrosis, provide information for risk stratification (58).

## CMR in Inflammatory Pericardial Disease

Although transthoracic echocardiography remains the first-line imaging modality to study patients with known or suspected pericardial inflammation, both computed tomography and CMR are considered important adjuvant imaging tools (20). As acute and chronic phases of pericardial inflammation may have a significant impact on the heart, a state-of-the art assessment of the pericardium necessitates a comprehensive approach.

In the acute phase of pericardial inflammation (“organizing pericarditis”), the pericardium is characterized by neovascularization, edema, thickening of the serous layers, and fibrin deposition with a variable amount of exudative and/or hemorrhagic fluid accumulating in the pericardial space (59). Chronic inflammation (“progressive sclerosing pericarditis”) is characterized by progressive fibrosis of the pericardium, fibrinous adhesion of the pericardial layers with or without pericardial exudation. Reduction of pericardial compliance and increased stiffness, with various grades of dystrophic calcification, may lead to constrictive physiology (“healed, organized fibrotic pericarditis”).

**FIGURE 9:**
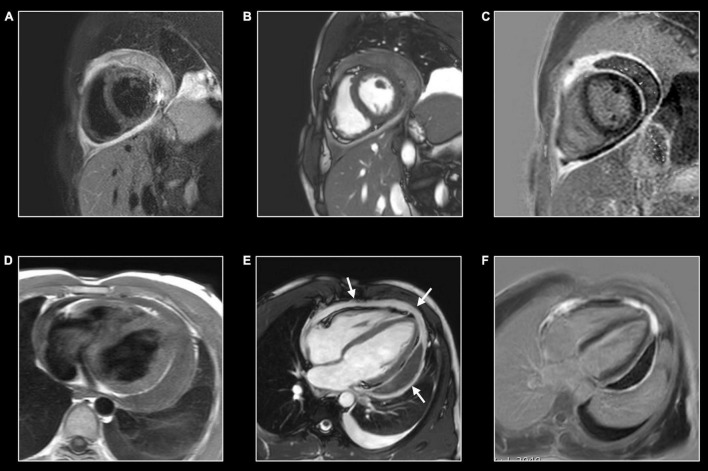
Hemorrhagic pericarditis. Twenty-six-year-old man presenting with hemorrhagic pericarditis of unknown origin. CMR shows important thickening of the pericardial layers **(A,D)** with localized pericardial effusion along the left lateral border **(B)**. T2w-imaging **(A)** allows to depict edema of the pericardial layers, while T1w imaging **(D)** is helpful to assess the thickness of the pericardial layers and to measure the maximal pericardial width. Strong enhancement of the inflamed pericardial layers which can be appreciated at LGE imaging in cardiac short-axis **(C)** and horizontal long-axis **(F)**. As bSFFP cine images were acquired post GBCA administration, the inflamed pericardial layers can be well appreciated too [arrows, **(E)**]. Note the presence of a moderate left-sided pleural effusion. Real-time cine imaging showed inspiratory early-diastolic septal flattening with increased septal shift, reflecting increased ventricular coupling caused by decreased compliance of the inflamed pericardial layers.

**FIGURE 10:**
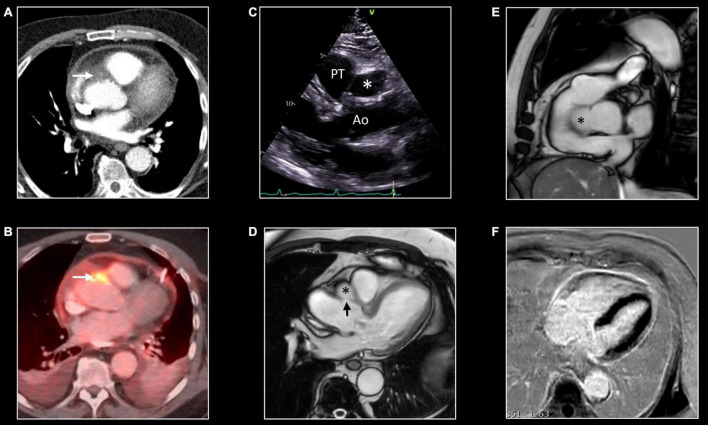
False aneurysm of the thoracic aorta. Eighty-one-year-old man with MSSA sepsis and infectious pericarditis complicated by false aneurysm formation of the thoracic aorta. CT and PET/CT performed after pericardial drainage showed thickening of the pericardial layers with enhancement at chest CT **(A)** and FDG uptake at PET/CT. Note the presence of a focus of strong FDG uptake [arrow, **(B)**] with infiltration of the epicardial fat [arrow, **(A)**] at CT. These findings were interpreted as inflammation of one of the pericardial sinuses. However, follow-up transthoracic echocardiography, 3 months later, showed echo-lucent structure [*,**(C)**] between the aorta (Ao) and pulmonary trunk (PT). CMR shows the presence of a small pseudo-aneurysm [*,**(D,E)**] in communication with the thoracic aorta at the level of the sinotubular junction [arrow, **(D)**]. Note the residual enhancement of the pericardial layers at LGE imaging, **(F)** reflecting residual pericardial inflammation.

Pericardial thickening is best imaged by dark-blood T1w imaging, while pericardial edema results in increased SI of the pericardium at T2w imaging (Figure 9) (20). Both T1w and cine imaging allow for the assessment (and if needed for the quantification) of pericardial effusion, while T1 mapping is promising to tissue characterize pericardial effusion. Although quantification of the pericardial effusion is possible, usually in clinical practice linear measures of the greatest distance between the two layers are used to estimate pericardial effusion severity (16). The normal pericardium is almost an avascular structure with consequent no or only mild contrast uptake. In case of inflammation, the post-contrast acquisition (LGE) shows pericardial enhancement as a result of pericardial vascularization and edema with a sensitivity of nearly 94% (60). A combination of LGE and T2w imaging may help determine the stage of inflammation. An intense LGE with a hyperintensity in T2w images reflects acute inflammation (Figure 9), whereas pericardial LGE with a normal T2 signal is suggestive of subacute pericarditis. Moreover, LGE assessment is a valuable tool for predicting the risk of and diagnosing recurrent pericarditis (61). Fibrous adhesion of the pericardial layers can be imaged by tagging sequences (20). Concomitant myocardial inflammation may be imaged by CMR as discussed in the previous paragraph. Rare complications such as post-pericarditis false aneurysm formation can be imaged adequately with CMR (Figure 10).

Morphologic features of constrictive pericarditis are pronounced thickening and irregularities of the pericardial layers. Calcification cannot be imaged by CMR but may be suspected in case of very low SI of the thickened pericardium on T1w and cine images (60). Thickening and calcifications occur more frequently in the right side and near the atrio-ventricular groove (62). However, up to 18% of patients with histologically proven constrictive pericarditis do not show a significantly increased thickness of the pericardium (63). Pericardial enhancement in the context of constrictive pericarditis is a sign of residual inflammation. Its detection is of crucial importance to guide inflammatory therapy and, possibly, to reverse the constrictive physiology. Hemodynamic consequences of constrictive pericarditis are: (a) dissociation between intrathoracic and intracardiac pressure, (b) increased ventricular coupling, (c) increased ventricular pressure with equalization of filling pressure in all four chambers.

Because of increased pericardial stiffness, impaired ventricular filling, and increased diastolic pressures, the right atrium, inferior vena cava, and hepatic veins are frequently dilated. Recently, mapping techniques have been proposed as a tool for imaging hepatic congestion, characterized by increased T1 and T2 relaxation times (64). Increased ventricular interdependence can be imaged by cine sequences showing early diastolic septal flattening or inversion (”septal bounce”). These abnormal septal movements are dramatically enhanced by respiratory variation. Consequently, real-time cine acquisition with deep inspiration and expiration are extremely useful especially in the differentiation of constrictive and restrictive physiology (19). Of notice, transient constriction is not uncommon in patients with effusive acute pericarditis (65).

## CMR in Systemic Vasculitis

**FIGURE 11:**
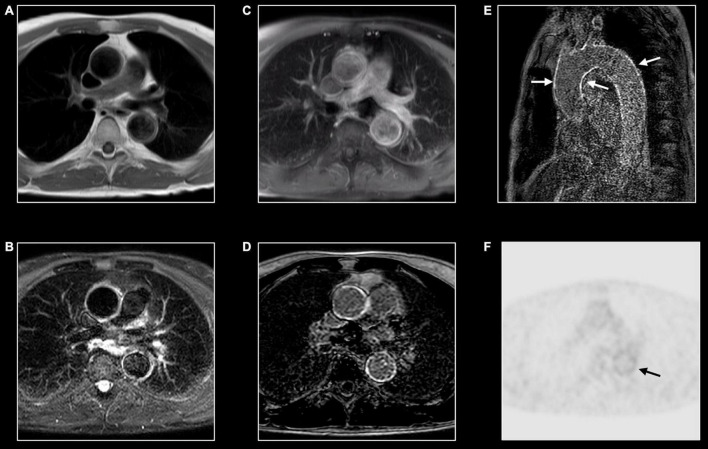
Immunoglobin G4-mediated aortitis in 57-year-old man. T1w imaging **(A)** shows mild dilatation of the proximal descending aorta with mild wall thickening. Hyperintense appearance of both ascending and descending aorta at T2w imaging **(B)** with contrast enhancement at post-contrast T1w imaging **(C)** and LGE imaging **(D)**. A long-axis view through the thoracic aorta nicely shows the diffuse wall enhancement [arrows, **(E)**]. FDG-PET shows mild to moderate tracer uptake [arrow, **(F)**] in the descending aorta [arrow, **(F)**].

Cardiovascular magnetic resonance is a versatile technique that provides information for the non-invasive assessment of primary vasculitides. Bright-blood bSSFP cine and dark-blood T1w images, acquired in the axial, sagittal, and coronal planes allow morphological assessment of great vessels providing information about the thickness and wall regularity (Figure 11) (15). T2w images with fat signal saturation permit the identification of vascular and peri-vascular tissue edema. Angiographic evaluation of the great and medium vessels can be achieved both with and without contrast administration. Bright-blood whole-heart, respiratory-navigated CMR provides 3D images of the aorta and coronary arteries without the need for contrast. Time-of-flight angiography (TOF) is a non-contrast technique useful for the assessment of intracranial and peripheral vessels. After GBCA administration, three-dimensional (3D) contrast-enhanced magnetic resonance angiography (CE-MRA) with 3D rendering, MIP and MPR reconstruction allow accurate vessel lumen evaluation (Figure 4) (15). T1w post-contrast acquisition provides information about post contrast enhancement.

Inflammation of the aorta and its main branches are characterized by common features as mural thickening (i.e., >2–3 mm is considered diagnostic for aortitis), wall, and periaortic soft tissue edema on T2w images, and post-contrast enhancement (Figure 11) (66). Layering thrombus and ulcerative process may be also encountered.

Distribution and associated features differ by etiology. Extracranial giant cell arteritis is the most frequent etiology of aortitis with classical MRI signatures previously described. Significant wall thickening with consequent stenosis and aneurysmatic dilatation characterize Takayasu arteritis with specific involvement of aorta and main branches, more frequently subclavian arteries (Figure 4) (67). Pulmonary artery involvement has been reported in up to 70% of cases but the real prevalence is still unknown (68). Coronary ostial stenosis may lead to ischemic myocardial lesions detected by CMR as subendocardial LGE with different grades of segmental or global systolic dysfunction. Coronary involvement is the most concerning complication of Kawasaki disease with coronary artery dilatation, stenosis, thrombus formation, and possible myocardial ischemic lesions (Figure 5) (69, 70).

Immunoglobulin G4–related aortitis is characterized by an aggressive course with intense periaortic involvement and acute aortic complications (e.g., intramural hematomas and aortic dissections) (Figure 11) (71). Behçet disease is a multi-systemic vasculitis whose hallmarks are oral and genital ulcerations, arthritis and ocular involvement. Both arterial and venous vascular systems can be affected with consequent stenosis and/or dilatation. While pericarditis is the most common cardiac manifestation, myocardial involvement has been reported in literature (66). CMR provides useful information in case of mycotic aneurysm, syphilitic and tuberculous aortitis showing diffuse, or more frequently focal, wall thickening, edema, and contrast enhancement with or without aneurysmatic dilatation (66). Medium and small-vessel vasculitis may also cause cardiovascular complications. Apart from the aforementioned Kawasaki disease, polyarteritis nodosa may cause inflammatory stenosis and aneurysmatic dilatation of medium-size arteries, including coronary arteries. As previously mentioned, in patients with eosinophilic granulomatosis with polyangiitis (the former Churg-Strauss syndrome) CMR can show myocardial involvement similarly to other eosinophilic myocarditis with diffuse subendocardial LGE, systolic dysfunction, and intraventricular thrombosis (72).

## Future Directions

**FIGURE 12:**
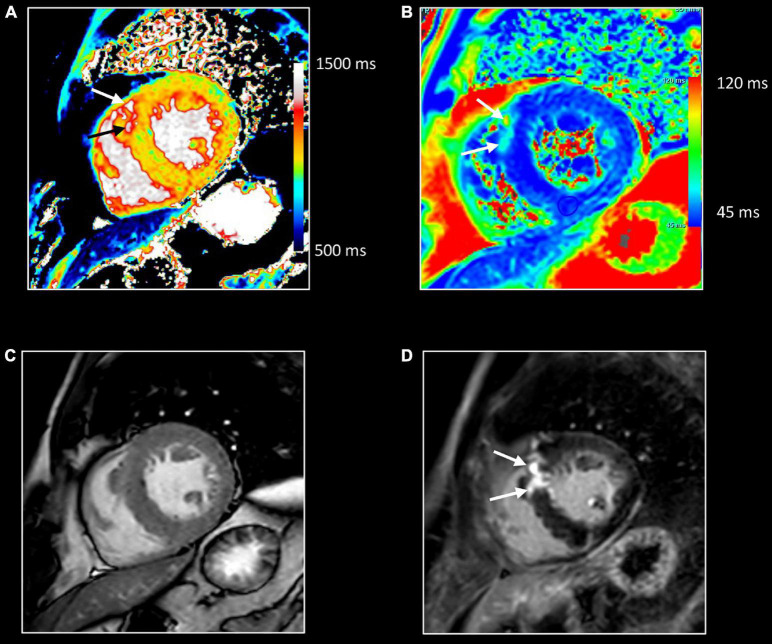
Myocardial inflammation in hypertrophic cardiomyopathy. Forty-seven-year-old man with familial hypertrophic cardiomyopathy (PRKAG2 mutation). All images were obtained in mid-ventricular short-axis. Thickened ventricular septum (max 20 mm) [cine image, **(C)**]. Focal small area of increased native T1 [arrows, **(A)**] and T2 values [arrows, **(B)**] in the thickened anteroseptal LV wall, closely corresponding to the area of myocardial fibrosis at LGE imaging [arrows, **(D)**]. These findings confirm the presence of concomitant myocardial edema in the fibrotic myocardium.

**FIGURE 13:**
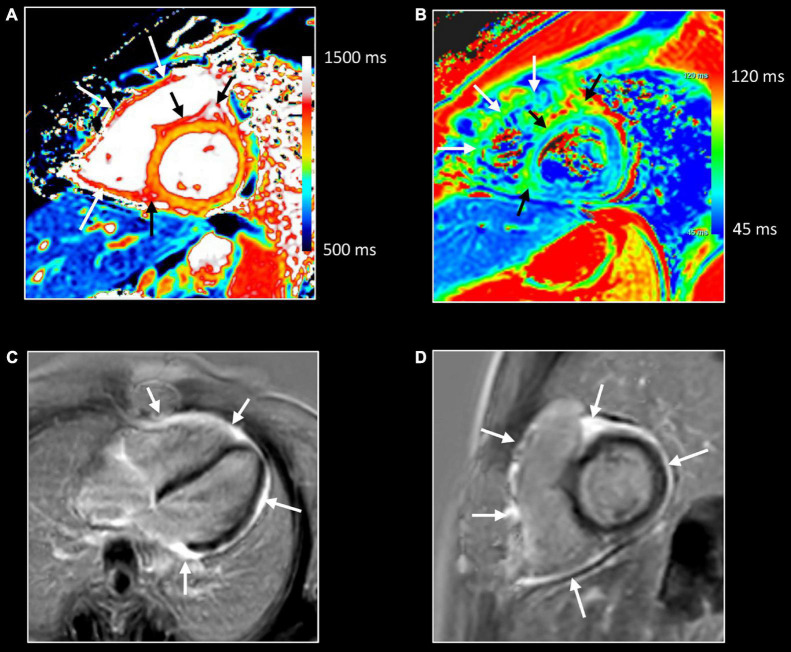
Allograft rejection. Twenty-two-year-old woman with history of univentricular heart, re-transplantation in 2021, and clinical evidence of acute rejection. CMR shows diffuse increase of native T1 **(A)** and T2 **(B)** myocardial values, most pronounced on the right side of the ventricular septum extending to the anterior/posterior atrioventricular groove [black arrows, **(A,B)**], and in the right ventricular (RV) wall [white arrows, **(A,B)**]. LGE imaging shows strong enhancement in RV wall, subepicardial left ventricular wall, and right side of the ventricular septum extending to the anterior/posterior atrioventricular groove [arrows, **(C,D)**]. Endomyocardial biopsy shows acute cellular rejection with diffuse extensive lymphocytic and eosinophilic inflammation [grade 3A (IHSLT 1990)–grade 2R (IHSLT 2004)], and myocardial fibrosis.

Inflammation has been proposed as a key factor in many other cardiovascular diseases, especially those characterized by myocardial injury. Different grades of myocardial inflammation have been demonstrated by endomyocardial biopsy in patients with sarcomeric hypertrophic cardiomyopathy and associated with MRI-proven myocardial fibrosis (Figure 12) (73). Evidence supporting the role of the inflammatory process in atherosclerosis accumulated in recent years. Moreover, activation of the inflammatory pathway was proven in patients with acute myocardial infarction not only in injured segments but also in areas of remote myocardium (74). Targeting inflammatory pathways with specific therapies was proven to improve LV remodeling in preclinical studies but failed in reaching significant outcomes in clinical ones (75). Recent evidence suggests the role of inflammation as a driver for phenotypic expression in patients with arrhythmogenic cardiomyopathy (76). Increased susceptibility to viral infection and immune activation may act on top of genetic predisposition. Moreover, myocarditis has been proposed as an additional criterion for arrhythmogenic cardiomyopathy (77). Further studies using non-invasive imaging for the assessment of inflammation in these settings are needed. As mentioned above, allograft rejection after heart transplant is a specific form of inflammatory myocardial disorder (9). Here too, CMR may add valuable information in addition to the endomyocardial biopsy (Figure 13).

18F-fluoro-deoxy-glucose (FDG) positron emission tomography (PET) is the gold standard for the detection of regions with high glucose uptake due to infection or malignancy. A localizing computer tomography (CT) provides anatomical information. In the context of CV inflammation, PET can be used for imaging cardiac device infection, cardiac sarcoidosis, vulnerable atherosclerotic plaque, and aortitis (Figures 8, 11) (78). The role of PET/CT in the detection of myocarditis has not been systematically investigated and evidence is limited to few reports (79). As for PET/CT, PET/MR provide simultaneously anatomical and metabolic information. Recent investigations showed the utility of hybrid FDG PET/MR in the detection and assessment of myocarditis (79). Abnormally increased myocardial uptake correlated well with the other established cardiac MR biomarkers. Moreover, PET may provide complementary information incrementing the sensitivity and specificity of MR in case of mild or borderline myocarditis or chronic inflammation (Figure 11) (80). Initial reports investigated the application of PET/MR in detecting aortic inflammation showing the feasibility, safety, and low radiological exposure (81).

Targeting specific inflammatory pathways is not possible with standard CMR. Iron oxide particle imaging allows identification of tissue macrophage infiltration (82). Ultra-small particles of iron oxide (USPIOs) or larger microparticles of iron oxide (MPIOs) are injected intravenously. After diffusion through capillary endothelia, they are taken up by phagocytic cells. Due to a strong paramagnetic effect of iron, areas of intense phagocytic infiltration are identified by low T1, T2, and T2* signals. Iron oxide particle magnetic resonance imaging has been used to detect active macrophage infiltration in atherosclerotic plaques (83) and in infarcted and remote myocardium in patients with chronic ischemic cardiomyopathy (82). On the other hand, the added value in detecting myocarditis has not been proven yet (82). The limited utility of iron particles can be explained considering that the lymphocytic signature is the most common inflammatory pathway in myocarditis.

Perfluorocarbons (^19^F) enhanced MRI is an alternative approach for imaging immune cells in cardiovascular disease (84). As iron oxide particles, biochemically inert nano-emulsions of perfluorocarbons are taken up by the macrophage/monocyte system. While they are widely used as contrast agents for preclinical applications, their translation in the potential clinical application needs further studies. Hyperpolarized (HP) MRI is a novel technology that improves the signal-to-noise ratio by increasing spin polarization of external substances. Using [1-^13^C] pyruvate HP probe, MRI can image the metabolic reprogramming of activated immune cells toward glycolysis (85). This immunometabolic reprogramming is shared by both phagocytic cells and lymphocytes, overcoming the limit of iron particle imaging in detecting lymphocytic inflammation. Another HP molecule, [1,4- ^13^C_2_] fumarate, allows targeting of active myocardial necrosis exploiting metabolic pathways activated in cells with the damaged cellular membrane (86). Although waiting for clinical translation, HP MRI is a promising tool for imaging inflammatory cardiovascular diseases.

## Conclusion

Inflammatory cardiomyopathy, pericarditis, and large vessels vasculitis still represent a challenge for physicians. Pathophysiological characteristics as vasodilation, exudation, leukocytes infiltration, cell damage, and fibrosis are used by CMR as diagnostic biomarkers. For this purpose, T2 weighted images, early and late gadolinium enhancement, and parametric mapping techniques are used. Future developments of CMR, as the assessment of the specific immune cell infiltration pathway, will provide deeper insight into cardiovascular inflammatory diseases.

## Author Contributions

DF, JB, and TD: conceptualization. DF: writing. JB and TD: supervision. JB: image selection. All authors contributed to the article and approved the submitted version.

## Conflict of Interest

The authors declare that the research was conducted in the absence of any commercial or financial relationships that could be construed as a potential conflict of interest.

## Publisher’s Note

All claims expressed in this article are solely those of the authors and do not necessarily represent those of their affiliated organizations, or those of the publisher, the editors and the reviewers. Any product that may be evaluated in this article, or claim that may be made by its manufacturer, is not guaranteed or endorsed by the publisher.
